# Network structure of non-suicidal self-injury, alcohol and food addiction, and motivation in bus drivers

**DOI:** 10.3389/fpsyt.2026.1795900

**Published:** 2026-05-14

**Authors:** Xun Song, Wen-wen Miao, Yang-ying Bu, Fang Wang, Hui Zheng, Yi-fei Li, Yi-hang Huang, Qiang Hu

**Affiliations:** 1 Unit of Psychiatry, Department of Public Health and Medicinal Administration, &Institute of Translational Medicine, Faculty of Health Sciences, University ofMacau, Macao, Macao SAR, China; 2 Centre for Cognitive and Brain Sciences, University ofMacau, Macao, Macao SAR, China; 3 Department of Psychiatry, Wuhu Hospital of Anding Hospital (The Fourth People’s Hospital of Wuhu), Wuhu, Anhui, China; 4 Shanghai Key Laboratory of Psychotic Disorders, Shanghai Mental Health Center, Shanghai Jiao Tong University School of Medicine, Shanghai, China; 5 Department of Computer Science, Sapienza University of Rome, Rome, Italy; 6 Department of Psychiatry, University of Cambridge, Cambridge, United Kingdom; 7 School of Mental Health and Psychological Sciences, Anhui Medical University, Hefei, Anhui, China

**Keywords:** alcohol addiction, bus drivers, food addiction, motivation to drink, non-suicidal self-injury (NSSI)

## Abstract

**Introduction:**

Professional bus drivers work in a safety-critical, high-demand context that may increase vulnerability to non-suicidal self-injury (NSSI) and addictive behaviors. However, the symptom-level mechanisms underlying this risk remain poorly understood, limiting the development of targeted, occupation-sensitive prevention and intervention strategies.

**Methods:**

Using cluster sampling, 1250 bus drivers were screened in southern Anhui (June 1–July 1, 2023). Participants completed standardized assessments, including a questionnaire based on the Diagnostic and Statistical Manual of Mental Disorders, Fifth Edition (DSM-5) proposed criteria for NSSI, the Alcohol Use Disorders Identification Test (AUDIT), the Yale Food Addiction Scale 2.0 (YFAS 2.0), and the Habit, Reward, and Fear Scale (HRFS). We estimated a regularized partial correlation network and constructed Bayesian directed acyclic graphs (DAGs) to examine directional relations.

**Results:**

In the regularized partial correlation network, Reward and Fear were the most central nodes, showing the highest levels of strength and expected influence. Alcohol addiction was positively associated with food addiction, NSSI, and motivation. In the DAG, Reward and Fear were positioned as putative upstream nodes while AUDIT as a terminal node. Motivation and food addiction symptoms showed direct edges to alcohol addiction, whereas NSSI showed a putative indirect link to AUDIT via YFAS.

**Conclusion:**

These results indicate structured interrelations among NSSI, alcohol addiction, food addiction, and motivation in bus drivers, and nominate Reward- and Fear-related processes as promising intervention targets. These insights offer a new perspective on the psychological network underlying risk behaviors in this occupational group and may inform strategies to improve mental health of transportation workers and public transportation safety.

## Introduction

Bus drivers constitute a safety-critical, passenger-facing service occupation characterized by demanding working conditions, including dense urban traffic and real-time hazard monitoring, high vigilance demands coupled with monotony, rigid timetables and route adherence, irregular/shift schedules with circadian disruption, crowd-management and customer-contact strain, constrained breaks, and high accountability for errors (1). Prior research has linked such occupational demands and related psychosocial strain to elevated risks of alcohol addiction, food addiction, and non-suicidal self-injury (NSSI) (2–4). These risk behaviors affect drivers’ health and may endanger public safety, but their underlying mechanisms remain insufficiently understood.

The Research Domain Criteria (RDoC) framework provides a useful tool for understanding these behaviors beyond traditional diagnostic categories (5). A Delphi consensus highlighted the role of positive valence system (reward valuation, expectancy, action selection, and reward learning), the sensorimotor system (habit formation), and the negative valence system (fear and loss), as central to addictive processes (6). These mechanisms are not limited to alcohol use but extend to compulsive eating and repetitive self-injurious acts, where reward-driven initiation, avoidance of aversive states, and habit consolidation appear to operate at different stages of behavioral persistence (6).

Building on this framework, clinicians and researchers are encouraged to move beyond the Diagnostic and Statistical Manual of Mental Disorders, Fifth Edition (DSM-5) diagnostic boundaries and instead appraise underlying features of psychopathology from a neuroscience-based perspective (7). The Habit, Reward, and Fear Scale (HRFS) was developed to assess three domains—positive valence (reward), sensorimotor (habit), and negative valence (fear/avoidance)—which have distinct neurobiological substrates and are thought to drive addictive and compulsive behaviors across multiple conditions (8). Early findings suggest that habitual motives are associated with broader severity across domains, while reward-related motives link more narrowly to impulsivity features (9). Although originally applied to alcohol use, these dimensions may equally capture motivational processes underlying food addiction and NSSI, providing a common metric to examine how reinforcement systems shape maladaptive behaviors across different domains (10).

Research has shown that the severity of alcohol dependence was linked to habitual drinking motives in drivers of alcohol addiction (8). Similarly, another study exploring emotional drivers of alcoholism reported that individuals often drink to seek rewards or to cope with fear (11). Research has also shown that NSSI frequently co-occurs with alcohol and substance abuse, and individuals may alternate between NSSI and high-risk drinking when underlying mechanisms remain unaddressed (12). In a review of the biological underpinnings of NSSI, the researchers noted that individuals who engage in NSSI exhibit heightened reward system activity in response to rewarding stimuli (13). Given the observed link between NSSI and alcohol addiction, it is plausible that drinking motives—particularly those related to reward—may also play a role in the development or maintenance of NSSI.

In addition, researchers reported that subjects with food addiction also have a higher lifetime prevalence of NSSI (14), and that those with food addiction also show dysfunction with similar characteristics to those of alcohol addicts (high impulsivity and low self-direction) (15), which are findings suggesting that there may be an association between the three, but few studies have mentioned it. Meanwhile, it has been pointed out that when food addicts are confronted with food, the reward circuit in the brain releases dopamine to give them a sense of pleasure and satisfaction, which in turn reinforces their cravings (16), which is also similar to the motivation for rewarding alcohol consumption. Given the potential links discussed above, drinking motives, particularly those related to reward, may play a key role in the development and interaction of these conditions.

However, there is a lack of studies examining the interplay among NSSI, alcohol addiction, food addiction, and motivation within bus drivers. To address this gap, network analysis offers a powerful means of mapping the structure and dynamics of symptom interactions by representing symptoms as nodes and their associations as edges, thereby identifying central features that may inform prevention and intervention strategies (17). Yet, while such models capture centrality, they cannot establish directionality; therefore, Bayesian networks using directed acyclic graphs (DAGs) can provide complementary insights by estimating conditional dependencies and generating hypotheses about potential directional relationships between symptoms (18).

In this study, we conducted network analysis, and Bayesian network modeling based on six dimensions derived from four standardized questionnaires to examine NSSI symptoms, alcohol addiction, food addiction, and motivation among 1,600 bus drivers. Our objectives included: (1) describing the relationships among NSSI, alcohol addiction, food addiction, and motivation in this occupational group, and (2) inferring potential directed pathways among these variables. By identifying central symptoms and activation sequences within the symptom networks, this study aims to provide novel insights for the early detection and targeted intervention of NSSI, alcohol addiction, and food addiction in bus drivers, ultimately contributing to improved occupational health and public transport safety.

## Methods

### Procedure

**Figure 1:**
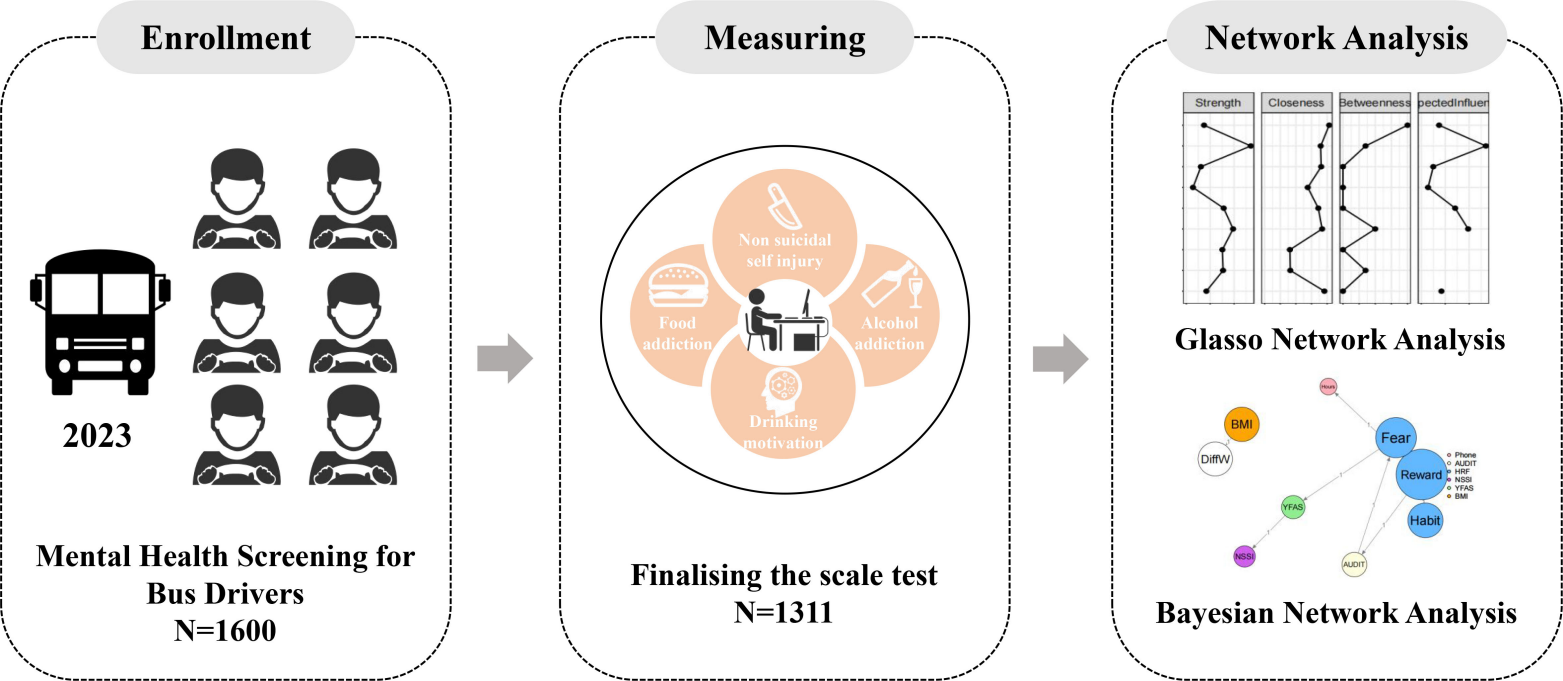
Flow chart of the study. Bus drivers working in the front line were recruited from the bus company, then they were invited to the computer room to fill in the questionnaire and finally analyzed by network analysis and Bayesian network in order to understand the relationship between their symptoms.

This study employed a cluster sampling method based on a mental health screening program organized by a local bus company in southern Anhui Province, China, between June 1 and July 1, 2023 (see **Figure 1** for the study flow). As part of this screening program, 1,600 frontline bus drivers were invited by the company to participate as a whole-cluster sample. Eligibility criteria included being between 18 and 60 years old and possessing sufficient literacy to understand and complete the self-report questionnaires. Drivers with a diagnosed mental illness were excluded from the study.

Of 1,600 invited, 1,311 completed the survey (completion rate 81.9%). Among them, 1,294 (98.7%) were male and 17 (1.3%) were female, with a mean age of 47.72 years (SD = 7.01). All participants were enrolled in the Mental Health Screening Program and completed the measures in a dedicated psychometric testing room under the supervision of trained mental health professionals. Prior to participation, each individual received a comprehensive explanation of the study’s purpose, procedures, potential benefits, and risks, and provided written informed consent.

This study was approved by the Ethics Committee of the Fourth People’s Hospital of Wuhu City, ethical approval number [2022]-KY-03.

### Measurement

To understand the NSSI behaviors among bus drivers, a self-report questionnaire was developed based on the diagnostic criteria for NSSI proposed in the DSM-5. Although these criteria were not included in the final version of the DSM-5 due to concerns regarding diagnostic reliability, subsequent psychometric studies have demonstrated that instruments based on this structure can validly identify individuals engaging in NSSI (19). This scale comprises six items corresponding to the six proposed DSM-5 criteria for NSSI. Specifically, items 1 through 5 assess symptom-related criteria, while item 6 evaluates exclusion criteria. The total symptom score is calculated by summing the scores of the first five items. Participants were classified as exhibiting NSSI behavior if their total symptom score was ≥5 and they did not meet the exclusion criteria.

To assess alcohol addiction among bus drivers, we employed the Alcohol Use Disorders Identification Test (AUDIT), an internationally recognized qualitative screening tool that has been widely validated for identifying individuals with alcohol dependence or hazardous drinking behaviors (20). The AUDIT consists of 10 items: items 1 to 8 are scored on a 5-point scale ranging from 0 (“never”) to 4 (“daily or almost daily”), while items 9 and 10 are scored 0, 2, or 4 based on responses ranging from “no” to “yes, during the past year.” The total score is calculated by summing the scores of all items, with a cutoff score of ≥8 indicating problematic alcohol use. In clinical interpretation, high scores on the first three items suggest serious hazardous drinking; elevated scores on items 4 to 6 reflect signs of alcohol dependence; and high scores on the final items are indicative of harmful alcohol use.

To assess food addiction among bus drivers, we utilized the Yale Food Addiction Scale 2.0 (YFAS 2.0), which was developed based on the DSM-5 criteria for substance use disorders. This scale is currently the only validated instrument specifically designed to assess food addiction, and prior research has demonstrated strong correlations between individual items and the overall scale score (21). The YFAS 2.0 consists of 35 self-report items corresponding to 11 symptom criteria and one diagnostic criterion. Each item has a predefined threshold; a response meeting the threshold is scored as 1, while responses that do not are scored as 0. For each of the 11 symptom criteria, if the sum of relevant item scores exceeds the threshold, the symptom is considered present and scored as 1; otherwise, it is scored as 0. The total symptom score is calculated by summing the 11 binary symptom scores, and the diagnostic score reflects whether the clinical significance criterion is met. A total symptom score of ≤1 or not meeting clinical significance indicates no food addiction; a score of 2–3 with clinical significance suggests mild food addiction; a score of 4–5 indicates moderate food addiction; and a score of ≥6 indicates severe food addiction.

To assess bus drivers’ motivation underlying additive behaviors, we employed a self-report scale adapted and translated from the Habit, Reward, and Fear Scale (HRFS) (8). The HRFS consists of 18 items originally designed to evaluate three motivational dimensions underlying drinking behavior: habit, reward, and fear. However, its structure makes it suitable for examining motivation across different addictive behaviors (10). Participants rated each item on a seven-point Likert scale ranging from “Strongly disagree” to “Strongly agree” based on their personal experiences. Specifically, items 3, 6, 7, 10, 14, and 16 assess habit-driven motivation; items 2, 4, 9, 12, 15, and 17 assess reward-based motivation; and items 1, 5, 8, 11, 13, and 18 assess fear-related drinking motivation. The total score for each dimension was calculated as the sum of the scores for its corresponding items.

### Data processing and statistics

This study used R (v4.2.3) for data preprocessing and network construction. To ensure data quality, we excluded 61 participants identified as careless responders based on reaction time (RT) screening using the “careless” package (using Mahalanobis distance across attention-check items; respondents above the 95th percentile were excluded). Our analytic sample comprised 1250 bus drivers (1,311 completers minus 61 exclusions).

### Network construction

We estimated the symptom network using the graphical least absolute shrinkage and selection operator (Glasso) method, which constructs a regularized partial correlation network with variables represented as nodes. Network estimation and visualization were conducted using the quickNet R package, which integrates the functionalities of qgraph, bootnet, and NCT. Four centrality indices were calculated: strength, closeness, betweenness, and expected influence. Strength refers to the sum of the absolute weights of all edges connected to a node, reflecting the extent of its direct connectivity. Closeness centrality is the inverse of the sum of the shortest paths from a given node to all other nodes, indicating the node’s indirect connectivity across the network. Betweenness centrality quantifies how frequently a node lies on the shortest path between two other nodes, reflecting its potential role in mediating interactions between other symptoms. Strength centrality is considered the most stable and reliable measure of a node’s importance. Expected influence is calculated as the sum of the raw (signed) edge weights connected to a node, accounting for both positive and negative associations, thereby providing a more nuanced index of node influence.

Network stability was assessed using correlation stability (CS) coefficients and 95% confidence intervals (CIs) of the edge weights, estimated via bootstrapping with the bootnet package in R. The CS coefficient indicates the proportion of cases that can be dropped while still maintaining a correlation of 0.70 or higher between the original and subset networks for centrality indices such as strength or bridge strength. A CS coefficient between 0.20 and 0.50 indicates moderate stability, 0.50 to 0.70 indicates good stability, and values above 0.70 reflect excellent stability. To evaluate the precision of edge weights, we generated 1,000 bootstrap samples to estimate 95% CIs. Wide or overlapping CIs suggest lower edge stability and greater estimation uncertainty.

### Bayesian network

We used the R package bnlearn to estimate and visualize the structure of a directed acyclic graph (DAG) representing the probabilistic directional relationships among variables in the network model. Structure learning was conducted using the Incremental Association Markov Blanket (IAMB) algorithm to identify an optimal Bayesian network structure. In this process, edges that appeared more frequently across bootstrap samples were assigned higher scores in terms of strength and directional probability. The initial network structure was refined by removing implausible arcs and reorienting illogical connections. A bootstrap procedure with 5,000 iterations was used to estimate arc strengths and improve the stability of the model. The resulting network was then averaged, retaining only those relationships with a strength above the 85th percentile. The final DAG illustrates the estimated directional probabilities among nodes and was visualized using qgraph.

## Results

### Symptom network analysis results

**Figure 2:**
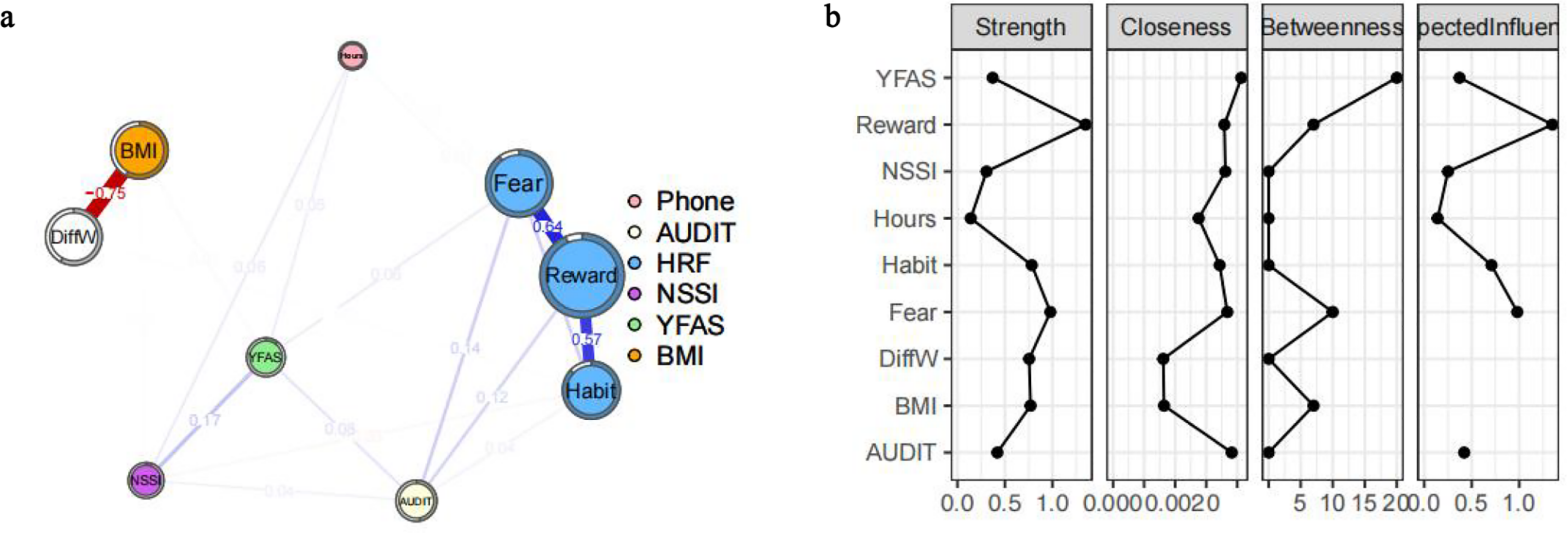
Symptom networks **(A)** Network structure of the Non-Suicidal Self-Injury (NSSI), Alcohol Dependence Identification Test (AUDIT), Yale Food Addiction Scale (YFAS), Motivation (Fear, Reward, and Habit), Daily Cell Phone Use (Phone), Body Mass Index (BMI), and Difference in Weight (DiffW). **(B)** Network structure of the NSSI. Positive correlations are indicated by blue borders and negative correlations by red borders. White circles indicate the expected value of each node. **(B)** Network centrality plots for nonsuicidal self-injury, alcohol addiction, food addiction, and motivation depict the strength, proximity, spacing, and expected impact (z-scores) of the selected variables in the current network.

To examine the network structure of symptoms related to NSSI, alcohol dependence, food addiction, and motivation among bus drivers, we conducted network analyses based on six dimensions derived from four standardized questionnaires. In addition to these psychological dimensions, three lifestyle and health-related variables—Daily Cell Phone Use (Phone), Body Mass Index (BMI), and Difference in Weight (DiffW)—were included in the network as covariates, resulting in a total of nine nodes. The estimated networks revealed distinct clustering patterns across symptoms and health behaviors (**Figure 2A**), with centrality indices displayed in **Figure 2B**. Notably, the symptom nodes from the NSSI, AUDIT, and YFAS scales were spatially distinct from those related to motivation, though strong within-domain associations were observed.

Among all nodes, Reward and Fear demonstrated the highest centrality in terms of strength and expected influence. Specifically, Reward showed Betweenness = 0.31, Closeness = 0.47, Strength = 1.34, and Expected Influence = 1.49; Fear showed Betweenness = 0.74, Closeness = 0.58, Strength = 0.98, and Expected Influence = 0.96. However, the correlation stability (CS) coefficient for betweenness was below 0.2, indicating poor stability; thus, betweenness results are not further interpreted. In contrast, the CS coefficients for strength, closeness, and expected influence all exceeded 0.7, reflecting excellent stability.

In terms of specific associations, Reward and Fear (edge weight = 0.64), as well as Reward and Habit (edge weight = 0.57), were most strongly connected within the HRF scale. The YFAS node showed positive associations with both the NSSI node and Fear, and NSSI, YFAS, and motivation were positively correlated with AUDIT. The integration of BMI, DiffW, and Phone into the network allowed for the observation of potential interactions between psychological symptoms and physical or behavioral health indicators.

Stability analyses confirmed the robustness of the network. **Supplementary Figure 1** shows the increasing average correlation of centrality metrics (betweenness, closeness, strength) with the original sample as the sample size increased. Bootstrapped difference tests using the bootnet package revealed significant differences in node strength, with additional details provided in **Supplementary Figures 2**, **3**.

### Bayesian network modeling results

**Figure 3:**
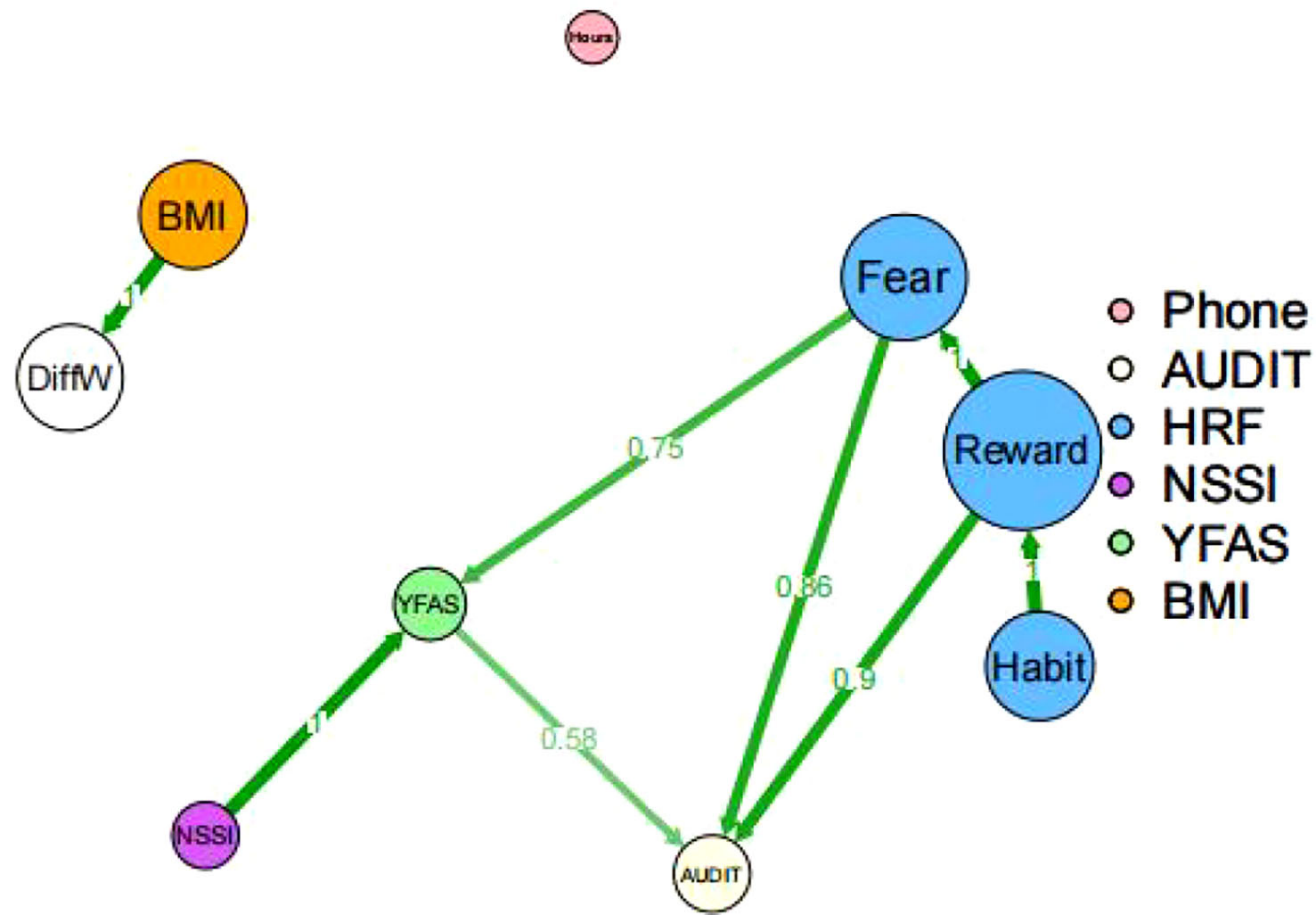
Directed acyclic graph (DAG) of Bayesian networks. Network structure of the Non-Suicidal Self-Injury (NSSI), Alcohol Dependence Identification Test (AUDIT), Yale Food Addiction Scale (YFAS), Motivation Processes (Fear, Reward, and Habit), Daily Cell Phone Usage (Phone), Body Mass Index (BMI), and Difference in Weight (DiffW). Green arrows indicate the estimated direction of edges in the averaged DAG. The thickness of the edges reflects the magnitude of the correlation.

As shown in **Figure 3**, the learned DAG comprised nine nodes and eight directed edges. The resulting structure revealed that Reward and Fear (dimensions of motivation) were positioned as upstream nodes, whereas AUDIT (alcohol dependence) was more likely to be located downstream. Within this network, Reward and Fear emerged as core nodes, influencing both YFAS (food addiction) and AUDIT directly.

Specifically, the AUDIT node was connected to YFAS (strength ≈ 0.6, direction ≈ 1), Reward (strength = 0.9, direction = 1), and Fear (strength ≈ 0.9, direction = 1), suggesting strong and unidirectional associations. Additionally, NSSI was directly linked to YFAS (strength = 1, direction = 1), showing that the structure is consistent with an indirect path via YFAS, which may indicate that food addiction serves as a mediator between NSSI and alcohol dependence. These findings suggest potential directed and transmission pathways, whereby motivational and behavioral factors contribute to the development of alcohol dependence among bus drivers. In particular, Reward, Fear, YFAS, and AUDIT formed a closely connected cluster, while NSSI influenced AUDIT indirectly via YFAS.

The DAG topology thus provides insight into the probabilistic dependencies and interactions among psychological symptoms and behaviors, offering a valuable framework for understanding the mechanisms underlying alcohol dependence in this population. The network structure was derived using the hill-climbing algorithm and Bayesian Information Criterion (BIC) scoring, with bootstrapping (n = 5,000) employed to ensure model stability. The model demonstrated satisfactory stability (mean loss = 22.77, SD = 0.71), and further details are presented in **Supplementary Figures 4**, **5**. Future studies are warranted to validate the symptom relationships suggested by this network structure.

## Discussion

This study is the first to examine the directed and undirected symptom network structure of NSSI, alcohol addiction, food addiction, and motivation among bus drivers. The network analysis showed that in bus drivers, NSSI, alcohol addiction, and food addiction were all connected to different motivational factors. The DAG suggested that alcohol addiction may be a downstream result, shaped by these motivational factors, and that NSSI may affect it indirectly through food addiction. These findings support prior hypotheses and provide new insights into the complex psychological mechanisms underlying addictive and self-injurious behaviors among bus drivers.

Alcohol addiction was more strongly linked to Reward and Fear motives than to Habit, with Reward and Fear emerging as the most prominent upstream nodes influencing alcohol addiction in the DAG. This indicates that drinking is primarily maintained by the pursuit of pleasurable outcomes and the avoidance of aversive states, rather than by habituation in this population. These findings align with the neurobiological models of addiction that addictive behaviors may activate the endogenous opioid system, enhancing euphoric pleasure while reducing psychological pain (22, 23). Moreover, the mesolimbic dopamine reward pathway has been widely recognized as a critical mechanism in the development and maintenance of addiction (24). However, Habit was identified as a direct upstream node influencing both Reward and Fear. This implies that, over time, motivational drivers such as reward seeking and fear avoidance may themselves become sustained through habituation. Prior research has similarly suggested that habituation is a long-term characteristic of alcohol addiction (9). From an intervention perspective, this indicates that strategies targeting reward sensitivity and emotion regulation may be particularly effective in the short term, while approaches focused on breaking habitual patterns may be essential for achieving long-term change.

Food addiction was associated only with Fear, with alcohol addiction identified as its downstream node and Fear and NSSI as upstream influences. This pattern suggests that compulsive eating in bus drivers may function primarily as a coping strategy to alleviate stress or negative emotional states. Prior research indicates that individuals with NSSI often experience persistent negative emotions and adverse life events (25), which can increase the use of high-calorie foods for emotional regulation. The sugar and fat content of such foods stimulate dopamine release, producing short-term mood improvement while fostering the development of food addiction (26). As palatable foods sensitize the brain’s reward circuitry, they may also heighten vulnerability to other addictive behaviors, including alcohol use (27). Moreover, the weight gain and metabolic disturbances associated with food addiction may lead individuals to seek alternative sources of gratification, such as alcohol consumption, particularly when food intake is consciously restricted. This substitution has been observed in post-bariatric surgery patients, where food addiction is frequently replaced by alcohol addiction (28).

NSSI was negatively associated with Habit, with food addiction identified as its downstream node. This finding suggests that self-injurious behavior in bus drivers is less likely to be maintained by habitual processes. Instead, NSSI may remain episodic or situational, emerging in response to acute stressors rather than becoming routinized (29). The negative association with Habit contrasts with the typical trajectory of addictive behaviors (3), where repetition usually fosters habituation, and may indicate that NSSI in this occupational group is governed by mechanisms distinct from those sustaining substance- or food-related addictions. The downstream link to food addiction further implies that, when NSSI does not persist as a habitual coping strategy, individuals may shift toward alternative maladaptive behaviors such as compulsive eating. Future research should clarify these dynamics by recruiting more representative samples of individuals with NSSI to better capture the motivational mechanisms involved.

### Limitations

This study has several limitations. First, all data were obtained through self-report measures, which may be subject to response biases such as social desirability or inaccurate recall. In addition, the requirement for sufficient literacy to complete the questionnaires may have introduced selection bias by excluding drivers with lower literacy levels, thereby limiting the representativeness of the sample. Second, the sample comprised bus drivers from a single geographic region and was predominantly male (only 17 women), which may limit the generalizability of the findings to broader populations. Third, the study did not include additional contextual variables such as mood states or significant life events, which may play an important role in the development of self-injurious and addictive behaviors (14). Fourth, the Bayesian network is only one way to estimate directional links (18). Because our data are cross-sectional, the arrows should be seen as hypothesis-generating rather than causal.

## Conclusion

In conclusion, the present study identified the network of NSSI, alcohol addiction, food addiction, and motivational factors among bus drivers, highlighting the central roles of Reward and Fear and identifying potential directional pathways between symptoms. These findings contribute specifically to occupational mental health and public safety by advancing understanding of how addictive and self-injurious behaviors are organized within a high-risk occupational group. This work may also offer guidance for developing targeted strategies to predict, prevent, and intervene in such behaviors.

## Data Availability

The raw data supporting the conclusions of this article will be made available by the authors, without undue reservation.
